# Overall Survival With Palbociclib and Aromatase Inhibitor Versus Aromatase Inhibitor Alone in Older Patients With HR+/HER2− Metastatic Breast Cancer

**DOI:** 10.1002/cam4.70719

**Published:** 2025-03-27

**Authors:** Adam M. Brufsky, Rickard Sandin, Stella Stergiopoulos, Connie Chen, Siddharth Karanth, Benjamin Li, Elizabeth Esterberg, Doris Makari, Sean D. Candrilli, Ravi K. Goyal, Hope S. Rugo

**Affiliations:** ^1^ University of Pittsburgh Pittsburgh Pennsylvania USA; ^2^ Pfizer AB Stockholm Sweden; ^3^ Pfizer Inc. New York New York USA; ^4^ RTI Health Solutions Research Triangle Park Raleigh North Carolina USA; ^5^ University of California San Francisco San Francisco California USA

**Keywords:** CDK4/6 inhibitor, HR+/HER2– metastatic breast cancer, older adults, overall survival, palbociclib, real‐world evidence, SEER

## Abstract

**Introduction:**

Cyclin‐dependent kinase 4/6 inhibitors (CDK4/6is) in combination with endocrine therapy are the current standard of care for first‐line (1L) treatment of hormone receptor–positive and human epidermal growth factor receptor 2–negative (HR+/HER2–) metastatic breast cancer (mBC). To investigate the effectiveness of palbociclib, the first‐in‐class CDK4/6i, plus an aromatase inhibitor (AI) in older patients, we compared overall survival (OS) in a Medicare population treated with 1L palbociclib + AI versus an AI alone.

**Methods:**

Patients aged ≥ 65 years who were diagnosed with de novo HR+/HER2– mBC from 2015 to 2019 were identified from the Surveillance, Epidemiology, and End Results (SEER)–linked Medicare database and were eligible if they initiated 1L palbociclib + AI or an AI alone. The primary endpoint was OS. Stabilized inverse probability of treatment weighting (sIPTW) was used to balance baseline patient characteristics.

**Results:**

Of 779 eligible patients, 296 received palbociclib + AI and 483 received AI alone as 1L treatment. After sIPTW, the median follow‐up was 23.1 months with palbociclib + AI and 18.2 months with AI alone. Adjusted median OS was longer with palbociclib + AI versus AI alone (sIPTW: 37.6 vs. 25.5 months, HR = 0.73 [95% CI, 0.59–0.91]). In multivariable Cox proportional hazards regression, patients treated with palbociclib + AI versus AI alone had a 39% lower risk of death (HR = 0.61 [95% CI, 0.48–0.77]).

**Conclusion:**

In routine US clinical practice, palbociclib + AI was associated with significantly prolonged OS versus AI alone in 1L treatment of patients aged ≥ 65 years with de novo HR+/HER2– mBC, adding to the growing body of evidence on the survival benefit of palbociclib + AI in this patient population.

**Trial Registration:**

ClinicalTrials.gov identifier: NCT06086340

## Graphical abstract

This real‐world study conducted with SEER‐Medicare data of patients aged 65 years or older with de novo HR+/HER2– metastatic breast cancer found that treatment with palbociclib in combination with an aromatase inhibitor (AI) was associated with significantly prolonged overall survival compared with AI alone.

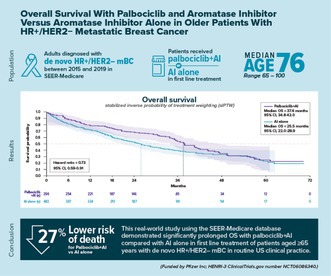

## 1 Introduction

Incidence rates of invasive breast cancer (BC) in the United States have increased since the mid‐2000s, and BC is the second‐leading cause of cancer‐related death in women [1]. As of 2019, patients with hormone receptor–positive/human epidermal growth factor receptor 2–negative (HR+/HER2–), the most common BC subtype, who develop distant metastases (metastatic breast cancer [mBC]) have a 5‐year relative survival rate of only 35.4%, although improvements seem to have occurred post 2015 [2, 3, 4]. Women aged ≥ 75 years have a higher risk of BC‐related death relative to younger women [5].

Nearly 70% of mBC is classified as HR+/HER2–, for which systemic endocrine therapy (ET) alone, including aromatase inhibitors (AIs), was the standard of care before 2015 [6, 7]. However, innovative targeted therapeutics, specifically cyclin‐dependent kinase 4/6 inhibitors (CDK4/6is), which were first introduced in 2015, altered the treatment paradigm. Palbociclib was the first CDK4/6i approved by the FDA in 2015, followed by ribociclib and abemaciclib in 2017, all based on similar primary end point progression‐free survival (PFS) benefits in their respective randomized clinical trials (RCTs): PALOMA‐1 and ‐2 [8, 9, 10], MONALEESA‐2 [11], and MONARCH‐3 [12]. Currently, CDK4/6is + ET are the standard of care for first line (1L) treatment of HR+/HER2– mBC [13, 14]. Despite ribociclib and abemaciclib entering the market, palbociclib still makes up a sizeable share of US CDK4/6i usage (data on file).

Long‐term results of the secondary endpoint, overall survival (OS), from RCTs with CDK4/6is + ET in 1L have been mixed. Although results from the PALOMA‐2 RCT showed significant improvement in PFS for patients receiving palbociclib plus letrozole over letrozole alone, no statistically significant effect was seen on OS [15]. Lack of a statistically significant OS gain was also seen with abemaciclib, while ribociclib showed significantly improved OS in respective Phase 3 RCTs [16, 17]. More recently, results from the randomized PARSIFAL‐LONG clinical trial evaluating the use of palbociclib in 1L mBC demonstrated a median overall survival (mOS) of 65 months in an endocrine‐sensitive patient population, which is more consistent with other 1L RCTs involving ribociclib and abemaciclib [18].

Despite the differences in OS from the trial setting, real‐world evidence (RWE) has indicated an OS advantage with CDK4/6is, including palbociclib. A recent study using the SEER database, with historical data prior to and after 2015, indicated a significant improvement in BC‐specific survival on a population level, potentially due to the introduction of CDK4/6is post 2015 specifically in the HR+/HER− population [19]. RWE is critical for understanding how therapies perform in routine clinical practice with diverse populations comprising patients with comorbidities, older age, or minority populations that are often excluded or underrepresented in RCTs. Few real‐world observational studies have assessed the effectiveness of 1L palbociclib plus an AI versus an AI alone in elderly US patients with HR+/HER2– mBC. In the P‐Reality‐X study using the Flatiron Health Analytic Database, palbociclib plus an AI was associated with improved OS and PFS overall and in older patients aged ≥ 65 years and ≥ 75 years, respectively [3, 4, 20]. Also, in a recent observational study using the Surveillance, Epidemiology, and End Results (SEER)–Medicare database [21, 22], Goyal et al. analyzed the early effect of the CDK4/6is on OS in patients aged ≥ 65 years with de novo HR+/HER2− mBC diagnosed in 2015–2017 across multiple lines of therapy. Overall, they reported that CDK4/6i plus ET versus ET alone was associated with improved OS [23].

Since the study by Goyal et al. [23] a new SEER‐Medicare dataset with two additional years of data has become available, allowing for longer follow‐up and further insight into the real‐world experiences of older CDK4/6i‐treated patients. This study (HENRI‐3: HR+/HER2− mBC characteristics and trends in real‐world survival in the United States for patients receiving palbociclib plus an AI vs. AI alone, NCT06086340) compared OS between patients with Medicare who are aged ≥ 65 years and have been diagnosed with de novo HR+/HER2– mBC treated with 1L palbociclib plus an AI versus an AI alone in routine practice settings.

## 2 Materials and Methods

### 2.1 Study Design and Data Source

This retrospective cohort study was performed using data from the SEER‐Medicare database, comprising two large, population–based data sources (SEER and Medicare). SEER registries include patient‐level demographic characteristics and clinical tumor data (e.g., stage, grade, HR/HER2 status) [21, 22, 24, 25, 26, 27, 28, 29]. SEER has been awarded the highest level of certification from the North American Association of Central Cancer Registries [30]. Linkage of SEER data with longitudinal healthcare utilization data from the administrative claims database for Medicare—which provides healthcare coverage for > 57 million individuals aged ≥ 65 years in the United States—captures detailed information about Medicare beneficiaries with cancer, including date of death, with over 95% of records validated by the Social Security Administration, and allows for retrospective “following” of patients, making SEER‐Medicare a unique data source to assess survival outcomes in a US population–based setting [21, 22, 31, 32, 33]. The 2023 release of SEER‐Medicare data captured ~35% of the total US population and includes Medicare patients aged ≥ 65 years with an incident cancer diagnosis in 1999–2019, with linked claims and survival data through 2020 [22, 34, 35].

### 2.2 Study Population

**FIGURE 1:**
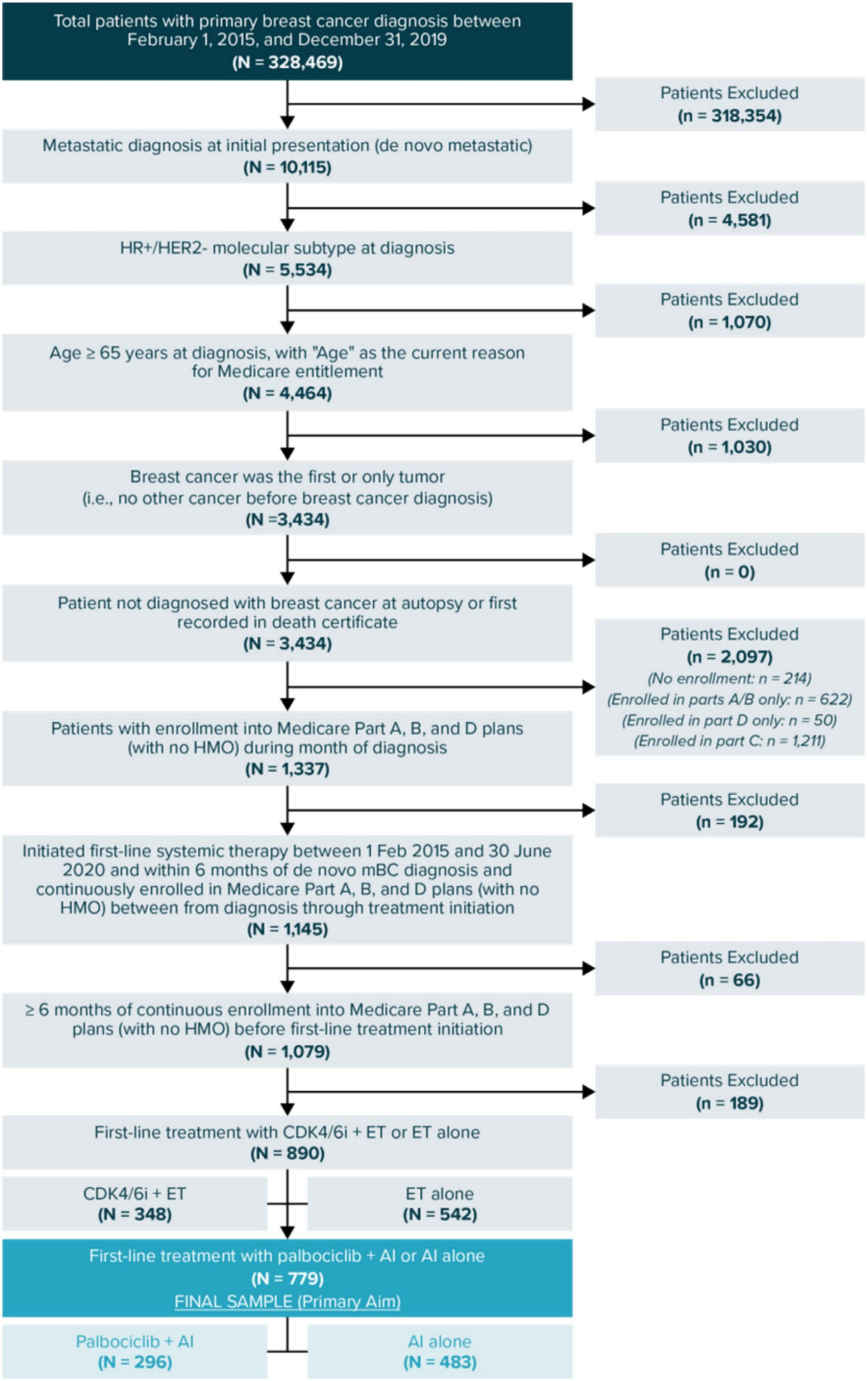
Patient selection flowchart.^a^ AI = aromatase inhibitor, CDK4/6i = cyclin‐dependent kinases 4 and 6 inhibitor, ET = endocrine therapy, HMO = health maintenance organization, HR+/HER2– = hormone receptor–positive and human epidermal growth factor receptor 2–negative, mBC = metastatic breast cancer. ^a^To ensure complete 1L therapy and healthcare encounter data, patients were required to maintain continuous enrollment in Medicare Parts A (inpatient care, hospital stays, care in a skilled nursing facility, hospice care, and some home health care), B (select healthcare provider [HCP] services, outpatient care, medical supplies, and preventative services), and D (prescription drugs) [40], with no HMO participation from the date of diagnosis until the index date and for ≥ 6 months before the index date. Patients were excluded if their mBC diagnosis was first recorded in a death certificate or at the time of autopsy.

The eligible study population included patients (female and male) diagnosed with mBC from February 1, 2015, to December 31, 2019. As neither database captures metastatic recurrences, this study focused exclusively on patients with de novo mBC [34, 36]. Eligible patients were aged ≥ 65 years with HR+/HER2– subtype, had mBC as their first and only tumor, initiated 1L systemic therapy with palbociclib + AI or an AI alone (i.e., index date) within 6 months of diagnosis, and had at least 6 months of continuous enrollment in Medicare Part A, B, and D plans before the index date [37, 38, 39]. To capture potential delays in treatment initiations, patients with a palbociclib claim within ±60 days of the start of AI treatment were included in the palbociclib + AI arm, where the first claim of either drug defined the index date. Figure 1 summarizes patient selection.

The follow‐up period was from the index date until death, Medicare disenrollment, enrollment in a health maintenance organization (HMO) plan due to lower data completeness in Medicare claims for HMO enrollees [41], or the date of database cutoff (December 31, 2020), whichever occurred first.

### 2.3 Study Measures

#### 2.3.1 Demographics and Clinical Characteristics

Baseline demographic characteristics included age; year of diagnosis; race; marital status; US community type, that is, rural–urban classification; low‐income subsidy coverage; and median household income. Baseline clinical characteristics included tumor grade and metastatic site involvement at diagnosis, and comorbidity burden assessed with the National Cancer Institute (NCI) comorbidity index [42, 43].

#### 2.3.2 Exposure Variables and Outcomes

The primary exposure was defined as a binary measure between 1L treatment type of palbociclib + AI versus an AI alone (anastrozole, letrozole, or exemestane). Second‐line (2L) treatments were also described. Treatment regimens were identified using generic drug names and Healthcare Common Procedure Coding System codes [44]. The primary outcome was OS, defined as time in months from the index date to the date of death for all causes. Patients alive at the end of follow‐up were censored in the survival analysis.

### 2.4 Statistical Analysis

Descriptive statistics were generated for all study variables, including means, standard deviations (SDs), medians, and interquartile ranges (IQRs) for continuous variables, and counts and percentages for categorical variables, as appropriate. OS was assessed using Kaplan–Meier (KM) analysis and multivariable methods. The primary method to balance differences in patient baseline characteristics was stabilized inverse probability of treatment weighting (sIPTW), a propensity score (PS)–based method frequently applied in observational studies to reduce potential confounding bias [45, 46, 47, 48, 49]. The PS (i.e., probability of assignment to treatment based on baseline covariates) was estimated using a multivariable logistic regression model adjusting for the baseline patient characteristics specified in the demographics and clinical characteristics section.

The covariate balance between the two treatment cohorts before and after sIPTW was assessed using standardized mean difference (SMD). An absolute SMD < 0.1 indicated negligible difference and was considered a good balance [48, 50]. In KM analyses, weighted mOS and 95% confidence intervals (CIs) were estimated, survival curves were drawn, and weighted landmark probabilities of events at various time points (e.g., 12 and 24 months) were estimated. sIPTW was applied to the Cox proportional hazards (CPH) model, and hazard ratio (HR) estimates and 95% CIs were derived. Variance was estimated using a robust variance estimation method to account for the weighted nature of the data [51].

In sensitivity analyses, OS was assessed using propensity score matching (PSM) and multivariable CPH regression methods, controlling for the same set of patient covariates used in sIPTW assessment [47]. For PSM, patients in the palbociclib + AI cohort were matched to those in the AI‐alone cohort using one‐to‐one matching with no replacement and the nearest neighbor method to match by closest PS (caliper of 0.01). Variance was estimated using a robust variance estimation method to account for clustering within paired sets.

Additional sensitivity analysis was conducted to assess any potential impact around the time of treatment initiation of the palbociclib and AI combination. Because our method allowed up to 60 days delay of start with palbociclib for the combination with AI therapy, additional sensitivity analyses using the same methods in our primary analyses were performed to explore the possible impact on OS estimates. First, the palbociclib + AI group index date was redefined as the date of palbociclib initiation (not necessarily the first drug in the combination) and second, the analysis was restricted to patients in either cohort who survived ≥ 60 days following the index date. Analyses were conducted using SAS statistical software, Version 9.4 (SAS Institute).

## 3 Results

### 3.1 Summary of Demographics and Clinical Characteristics

A total of 779 patients were eligible (Figure 1): 296 (38.0%) received palbociclib + AI (median age = 73 years [IQR = 10]) and 483 (62.0%) received an AI alone (median age = 78 years [IQR = 13]). Table 1 presents patient baseline demographic and clinical characteristics (Table S1 presents additional baseline comorbidities and clinical characteristics including locoregional therapy). Balance (SMD < 0.1) was achieved for all observed characteristics between the treatment groups after sIPTW and PSM (Table 1). Before sIPTW, the median time from mBC diagnosis to 1L therapy initiation was 44.5 days (IQR = 28.5) in the palbociclib + AI cohort and 47 days (IQR = 39) in the AI‐alone cohort (Figure S1A). The median time from AI initiation to palbociclib initiation in the palbociclib + AI cohort was 8 days (IQR = 22; Figure S1B). After sIPTW adjustment, the median length of follow‐up was 23.1 months (IQR = 25.7) in the palbociclib + AI cohort and 18.2 months (IQR = 24.3) in the AI‐alone cohort, a difference partly explained by differences in death events.

**TABLE 1:** Patient demographic and clinical characteristics before and after sIPTW‐weighting and PS matching.

	Before PS adjustment					After sIPTW‐weighting					After PS matching				
	1L palbociclib + AI		1L AI alone		SMD	1L palbociclib + AI		1L AI alone		SMD	1L palbociclib + AI		1L AI alone		SMD
	*n*	%	*n*	%		*n*	%	*n*	%		*n*	%	*n*	%	
Demographic characteristics															
All patients	296	100	483	100		296^a^	100	482^a^	100		244	100	244	100	
Age at initial diagnosis, years															
Median	73.0		78.0			76.0		76.0			74.0		74.0		
Mean (SD)	74.2 (6.4)		78.0 (7.8)			76.1 (7.0)		76.7 (7.7)			74.9 (6.6)		75.4 (7.3)		
Age group															
65–69	86	29.1	86	17.8	0.27	67	22.5	107	22.2	**0.01**	64	26.2	61	25.0	**0.03**
70–74	84	28.4	91	18.8	0.23	66	22.2	106	22.0	**0.01**	64	26.2	67	27.5	**0.03**
75–79	67	22.6	101	20.9	**0.04**	64	21.6	105	21.7	**0.00**	57	23.4	56	23.0	**0.01**
≥ 80	59	19.9	205	42.4	0.50	100	33.7	165	34.1	**0.01**	59	24.2	60	24.6	**0.01**
Race—recategorized															
Non‐White^c^	40	13.5	66	13.7	**0.00**	37	12.6	64	13.3	**0.02**	32	13.1	35	14.3	**0.04**
White	256	86.5	417	86.3		259	87.4	418	86.7		212	86.9	209	85.7	
Year of mBC diagnosis															
2015	43	14.5	89	18.4	0.11	46	15.4	80	16.6	**0.03**	39	16.0	39	16.0	**0.00**
2016	39	13.2	105	21.7	0.23	54	18.2	90	18.6	**0.01**	36	14.8	29	11.9	**0.08**
2017	74	25.0	88	18.2	0.17	65	21.9	102	21.2	**0.02**	59	24.2	58	23.8	**0.01**
2018	62	20.9	118	24.4	**0.08**	64	21.8	109	22.6	**0.02**	52	21.3	55	22.5	**0.03**
2019	78	26.4	83	17.2	0.22	67	22.8	102	21.1	**0.04**	58	23.8	63	25.8	**0.05**
Marital status at initial diagnosis—recategorized															
Single	39	13.2	61	12.6	**0.02**	38	13.0	61	12.7	**0.01**	N/A	15.2	31	12.7	**0.07**
Married or unmarried living with domestic partner	128	43.2	149	30.8	0.26	101	34.1	168	34.9	**0.02**	93	38.1	95	38.9	**0.02**
Divorced/separated/widowed	118	39.9	240	49.7	0.20	141	47.6	226	46.8	**0.02**	104	42.6	107	43.9	**0.02**
Unknown	11	3.7	33	6.8	0.14	16	5.4	27	5.6	**0.01**	N/A	4.1	11	4.5	**0.02**
Geographic status of residence															
Large urban	232	78.4	351	72.7	0.13	221	74.6	362	75.2	**0.01**	188	77.0	186	76.2	**0.02**
Small urban	50	16.9	88	18.2	**0.03**	48	16.1	83	17.2	**0.03**	42	17.2	40	16.4	**0.02**
Rural	14	4.7	44	9.1	0.17	28	9.3	37	7.7	**0.06**	14	5.7	18	7.4	**0.06**
Median household income															
Quartile 1 (< $42,881)	72	24.3	122	25.3	**0.02**	80	27.0	120	25.0	**0.05**	61	25.0	55	22.5	**0.06**
Quartile 2 ($42,881–$57,963)	66	22.3	129	26.7	**0.10**	70	23.6	122	25.2	**0.04**	60	24.6	60	24.6	**0.00**
Quartile 3 ($57,964–$84,595)	68	23.0	126	26.1	**0.07**	73	24.6	122	25.2	**0.02**	59	24.2	64	26.2	**0.05**
Quartile 4 ($84,596+)	90	30.4	106	21.9	0.19	73	24.8	118	24.5	**0.01**	64	26.2	65	26.6	**0.01**
Low‐income subsidy (LIS) coverage at any time during the 6‐month baseline period															
Any LIS coverage	82	27.7	153	31.7	**0.09**	90	30.3	144	29.9	**0.01**	70	28.7	71	29.1	**0.01**
No LIS coverage	214	72.3	330	68.3		206	69.7	338	70.1		174	71.3	173	70.9	
Clinical characteristics															
All patients	296	100	483	100		296^a^	100	482^a^	100		244	100	244	100	
Median follow‐up, months	23.9		18.2			23.1		18.2			24.7		18.3		
Q1, Q3	14.3, 39.5		7.1, 32.0			11.9, 37.6		7.7, 32.0			40.4, 13.5		8.1, 31.8		
Tumor grade at initial diagnosis															
1 (well differentiated)	31	10.5	57	11.8	**0.04**	32	10.9	57	11.8	**0.03**	29	11.9	25	10.2	**0.05**
2 (moderately differentiated)	135	45.6	204	42.2	**0.07**	130	44	210	43.5	**0.01**	111	45.5	105	43	**0.05**
3 (poorly differentiated)	61	20.6	91	18.8	**0.04**	57	19.3	92	19.1	**0.00**	42	17.2	47	19.3	**0.05**
Unknown	69	23.3	131	27.1	**0.09**	77	25.9	124	25.6	**0.01**	62	25.4	67	27.5	**0.05**
Metastatic site involved at initial diagnosis															
Bone	233	78.7	355	73.5	0.12	224	75.8	366	75.9	**0.00**	192	78.7	185	75.8	**0.07**
Brain	12	4.1	21	4.3	**0.01**	11	3.8	20	4.1	**0.01**	N/A	N/A	N/A	N/A	**0.04**
Liver	40	13.5	65	13.5	**0.00**	43	14.4	67	13.8	**0.02**	34	13.9	32	13.1	**0.02**
Lung	78	26.4	143	29.6	**0.07**	81	27.4	135	28	**0.01**	66	27	63	25.8	**0.03**
NCI comorbidity index score category^b^															
0	154	52	198	41	0.22	135	45.5	217	45	**0.01**	117	48	120	49.2	**0.02**
> 0–1	111	37.5	190	39.3	**0.04**	111	37.6	186	38.7	**0.02**	96	39.3	91	37.3	**0.04**
> 1	31	10.5	95	19.7	0.26	50	16.8	79	16.3	**0.01**	31	12.7	33	13.5	**0.02**
Median NCI score at index date	0.0		0.3			0.3		0.3			0.2		0.1		

*Note:* In compliance with the SEER‐Medicare Data Use Agreement, groups with frequencies N/A must be suppressed; therefore, data in some patient groups are collapsed for reporting or suppressed by marking as N/A to prevent derivation of the cell size below 11. Bold indicates covariate balance between two treatment cohorts was achieved (SMD < 0.10).

Abbreviations: 1L = first line, AI = aromatase inhibitor, ASMD = absolute standardized mean difference, mBC = metastatic breast cancer, N/A = not available due to low cell sizes, NCI = National Cancer Institute, PS = propensity score, SD = standard deviation, SEER = Surveillance, Epidemiology, and End Results, sIPTW = stabilized inverse probability of treatment weighting, SMD = standardized mean difference.

^a^ Sum of weighted frequencies.

^b^ Measured within 6 months before 1L therapy initiation; individual comorbidities are reported in Table S1.

^c^ Includes Black, Asian, and Native American/Pacific Islanders patients.

### 3.2 Overall Survival

**FIGURE 2:**
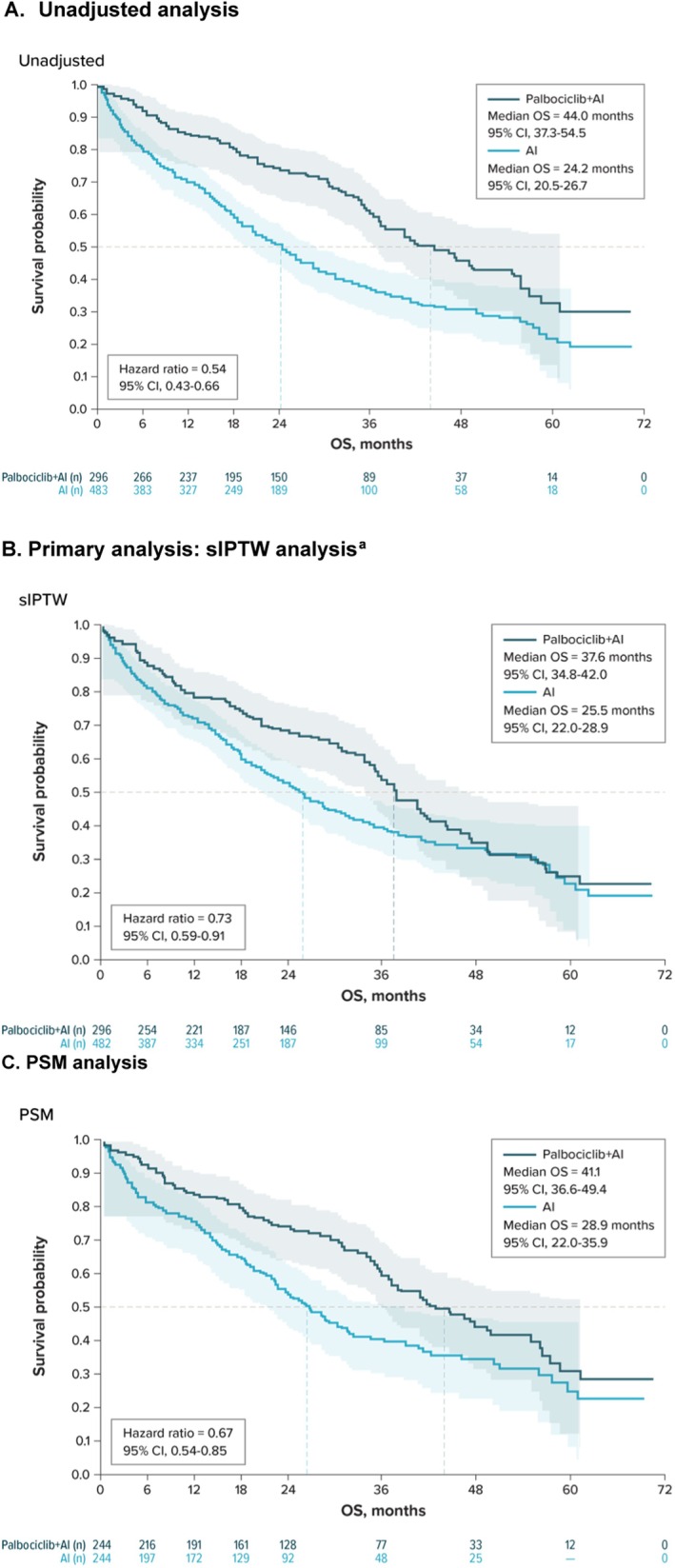
KM analysis of overall survival. (A) Unadjusted analysis. (B) sIPTW analysis^a^ (Primary analysis). (C) PSM analysis. AI = aromatase inhibitor, CI = confidence interval, KM = Kaplan–Meier, OS = overall survival, PSM = propensity score matching; sIPTW = stabilized inverse probability of treatment weighting. ^a^In the sIPTW analysis, there was a sign of potential violation of the proportional hazard assumption for the treatment type: the Schoenfeld residuals test was significant; however, an interaction of treatment type with the log of time was found to be not significant.

In the unadjusted KM analysis, mOS was 44.0 months (95% CI, 37.3–54.5) for the palbociclib + AI group versus 24.2 months (95% CI, 20.5–26.7) for the AI‐alone group (unadjusted HR = 0.54 [95% CI, 0.43–0.66]) (Figure 2A, Table 2). In the primary sIPTW‐adjusted analysis, mOS was 37.6 months (95% CI, 34.8–42.0) for the palbociclib + AI group versus 25.5 months (95% CI, 22.0–28.9) for the AI‐alone group (HR = 0.73 [95% CI, 0.59–0.91]) (Figure 2B, Table 2). OS rates are provided in Table 2.

**TABLE 2:** Overall survival (OS) in the unadjusted, sIPTW, and PSM analyses.

	Unadjusted analysis				sIPTW analysis^b^				PSM analysis			
	1L palbociclib + AI		1L AI alone		1L palbociclib + AI		1L AI alone		1L palbociclib + AI		1L AI alone	
Total patients, *n* (%)	296	(100)	483	(100)	296	(100)	482	(100)	244	(100)	244	(100)
Patients with event, *n* (%)^a^	119	(40.2)	298	(61.7)	146	(49.3)	280	(58.2)	107	(43.9)	129	(52.9)
Patients censored, *n* (%)	177	(59.8)	185	(38.3)	150	(50.7)	202	(41.8)	137	(56.1)	115	(47.1)
KM estimates												
OS time, median (95% CI), months	**44**	**(37.3–54.5)**	**24.2**	**(20.5–26.7)**	**37.6**	**(34.8–42.0)**	**25.5**	**(22.0–28.9)**	**41.1**	**(36.6–49.4)**	**28.9**	**(22.0–35.9)**
OS rate, % (95% CI)												
6 months	91.8	(88.0–94.4)	80.0	(76.2–83.4)	88.2	(83.3–91.7)	81.5	(77.5–84.8)	90.8	(86.4–93.9)	81.5	(76.0–85.8)
12 months	84.8	(80.1–88.4)	70.1	(65.8–74.0)	78.9	(73.1–83.5)	72.2	(67.7–76.1)	82.8	(77.3–87.0)	74.0	(67.9–79.0)
18 months	80.5	(75.4–84.7)	58.8	(54.2–63.1)	74.7	(68.6–79.8)	60.9	(56.1–65.5)	79.0	(73.2–83.7)	63.4	(56.8–69.2)
24 months	73.9	(68.1–78.8)	50.3	(45.6–54.9)	68.1	(61.6–73.8)	52.5	(47.5–57.3)	73.3	(66.9–78.7)	54.4	(47.5–60.8)
36 months	61.0	(54.1–67.3)	37.2	(32.4–42.0)	54.4	(46.8–61.3)	39.5	(34.3–44.6)	59.4	(51.8–66.1)	42.4	(35.1–49.6)
48 months	45.6	(37.5–53.3)	30.9	(26.0–35.9)	35.2	(27.4–43.1)	33.5	(28.2–38.9)	42.5	(34.1–50.7)	39.3	(31.7–46.7)
60 months	32.6	(22.8–42.7)	21.9	(16.2–28.2)	24.7	(16.6–33.7)	23.1	(16.7–30.1)	30.2	(20.3–40.7)	28.3	(18.8–38.5)
Univariate Cox regression												
Hazard ratio (95% CI)	**0.54**	**(0.43–0.66)**			**0.73**	**(0.59–0.91)**			**0.67**	**(0.54–0.85)**		

*Note:* median OS values in table 2 were bolded to indicate no overlap in the 95% confidence intervals. Hazard ratio in table 2 was bolded to indicate that 1 is not in any of the the 95% confidence interval. Recommend here also bolding median OS and Hazard ratio and not just the values.

Abbreviations: 1L = first line, AI = aromatase inhibitor, CI = confidence interval, KM = Kaplan–Meier, OS = overall survival, PSM = propensity score matching, sIPTW = stabilized inverse probability treatment weighting.

^a^ All‐cause death.

^b^ Primary analysis method.

Sensitivity analyses confirmed an associated OS benefit with palbociclib + AI versus AI alone. In the PSM supplemental analysis, mOS was 41.1 months (95% CI, 36.6–49.4) for palbociclib + AI group versus 28.9 months (95% CI, 22.0–35.9) for AI alone (HR = 0.67 [95% CI, 0.54–0.85]) (Figure 2C, Table 2). In the multivariable Cox regression analysis, palbociclib + AI was associated with a 39% lower risk of death than AI alone (HR = 0.61 [95% CI, 0.48–0.77]).

**FIGURE 3:**
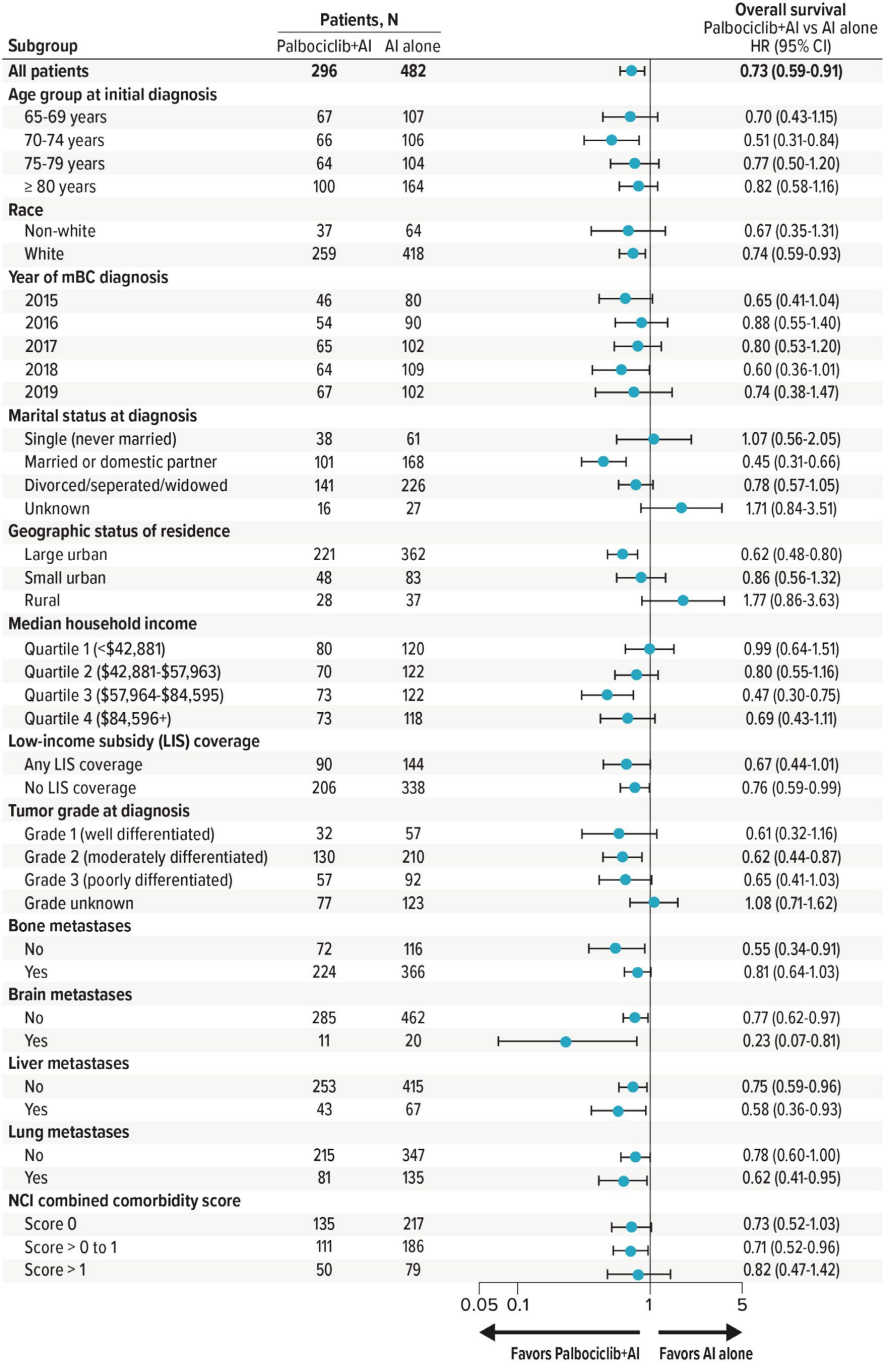
Forest plot of sIPTW‐adjusted overall survival by subgroups. AI = aromatase inhibitor, CI = confidence interval, HR = hazard ratio, mBC = metastatic breast cancer, NCI = National Cancer Institute, sIPTW = stabilized inverse probability of treatment weighting.

HRs for OS derived from sIPTW‐based analyses across most subgroups—including patients with liver metastases (HR = 0.58 [95% CI, 0.36–0.93]) and lung metastases (HR = 0.62 [95% CI, 0.41–0.95])—consistently showed an OS benefit with palbociclib + AI therapy versus an AI alone (Figure 3), although sample sizes were smaller at 110 and 216 total patients, respectively. The findings of sensitivity analyses based on redefining the index date as the date of palbociclib initiation and restricting analyses to patients who survived ≥ 60 days following the index date, respectively, were consistent with the primary results (HR = 0.74 [95% CI, 0.60–0.93] and HR = 0.75 [95% CI, 0.59–0.94], respectively), indicating that study outcomes were stable (Figures S2 and S3).

### 3.3 Subsequent Systemic Therapies

Following 1L therapy, 43.9% (*n* = 130) of patients in the palbociclib + AI cohort and 44.5% (*n* = 215) in the AI‐alone cohort received 2L therapy (Table 3). At study cutoff, more patients were still on 1L treatment in the palbociclib + AI cohort (37.2%) than in the AI‐alone cohort (19.0%), while more patients in the AI‐alone cohort did not initiate 2L therapy (36.4%) compared with the palbociclib + AI cohort (18.9%) (Table 3).

**TABLE 3:** Subsequent treatments received after 1L therapy.

Treatment category	*n*	%
1L palbociclib + AI^a^	**296**	**100.0**
Any 2L treatment^b^ (*n*, row %)	**130**	**43.9**
Endocrine therapy	112	86.2
Fulvestrant	82	63.1
Letrozole	21	16.2
Other	19	14.6
CDK4/6i	44	33.8
Palbociclib	33	25.4
Other	11	7.7
Chemotherapy	17	13.1
mTOR inhibitor	18	13.8
Combination therapy received in 2L	**70**	**53.9**
1L ongoing	**110**	**37.2**
No 2L treatment	**56**	**18.9**
Discontinued 1L due to death	40	13.5
Discontinued 1L for reason other than death	16	5.4
1L AI alone^a^	**483**	**100.0**
Any 2L treatment^b^ (*n*, row %)	**215**	**44.5**
Endocrine therapy	174	80.9
Fulvestrant	92	42.8
Exemestane	18	8.4
Letrozole	50	23.3
Anastrozole	44	20.5
Tamoxifen	13	6.0
CDK4/6i	98	45.6
Palbociclib	75	34.9
Abemaciclib	15	7.0
Chemotherapy	20	9.3
Combination therapy received in 2L	**105**	**48.8**
1L ongoing	**92**	**19.0**
No 2L treatment	**176**	**36.4**
Discontinued 1L due to death	138	28.6
Discontinued 1L for reason other than death	38	7.9

*Note:* In compliance with the SEER‐Medicare Data Use Agreement, groups with frequencies < 11 must be suppressed; therefore, data in some patient groups are collapsed for reporting. In Table 3, the bolding indicates either a subtotal or total. So for example: under 1L palbociclib + AI. The total = 296 pts. Any 2L Tx (130) + 1L ongoing (110) + No 2L Tx (56) = 296 pts.

Abbreviations: 1L = first line, 2L = second line, AI = aromatase inhibitor, CDK4/6i = cyclin‐dependent kinases 4/6 inhibitor, mTOR = mammalian target of rapamycin.

^a^ Switching between AI does not advance the line of therapy.

^b^ 2L treatments could include combinations of therapeutic agents. The most frequent 2L combinations for the 1L palbociclib + AI cohort were palbociclib + fulvestrant, everolimus (mTOR inhibitor) + AI, and everolimus + fulvestrant. The most frequent combinations for the 1L AI‐alone cohort were palbociclib + AI, palbociclib + fulvestrant, and fulvestrant + AI.

In the palbociclib + AI cohort receiving 2L therapy, 86.2% received ET (primarily fulvestrant [63.1%]), 33.8% received a CDK4/6i, and 13.1% received chemotherapy. In the AI‐alone cohort, 80.9% received ET (primarily fulvestrant [42.8%]), 45.6% received a CDK4/6i, and 9.3% received chemotherapy. Combination therapy in the 2L setting was common in both cohorts (palbociclib + AI [53.9%] and AI alone [48.8%]), which frequently included palbociclib.

## 4 Discussion

In this real‐world, population–based study using the SEER‐Medicare database, we found that treatment with palbociclib was associated with an OS benefit in patients aged ≥ 65 years with de novo HR+/HER2– mBC. Primary analysis using sIPTW showed a statistically significant 27% reduction in the risk of all‐cause death (HR = 0.73 [95% CI, 0.59–0.91]) for those receiving 1L palbociclib + AI versus an AI alone. Sensitivity analyses using PSM and Cox regression analyses, and the two additional sensitivity analyses assessing any potential impact around the time of treatment initiation of the palbociclib and AI combination, demonstrated consistency in the OS benefit of palbociclib + AI versus an AI alone. While limited by small sample sizes, the survival benefit was also seen in most subgroups, notably those with liver or lung metastases. A considerable proportion of patients in both cohorts were subsequently treated with a CDK4/6i in the 2L (33.8% for the palbociclib + AI cohort and, as expected, more frequently in the AI‐alone cohort [45.6%]). There were also almost twice as many patients still on 1L palbociclib + AI (37.2%) versus an AI alone (19.0%) at study cutoff. Overall, these findings support the use of palbociclib in older adults with de novo HR+/HER2– mBC.

Previous clinical trials have assessed the benefit of 1L palbociclib with an AI in HR+/HER2– mBC; the Phase 2 PALOMA‐1 [8, 52] and Phase 3 PALOMA‐2 [9, 15] trials both demonstrated significantly improved PFS (primary endpoint) with 1L palbociclib and letrozole versus letrozole alone. Results from the secondary OS endpoint of PALOMA‐2 were not statistically significant, including in patients aged ≥ 65 years [15]. Results from the PARSIFAL‐LONG RCT demonstrated an mOS of 65 months, in line with mOS results from 1L mBC RCTs with other CDK 4/6is [18].

RWE helps advance the understanding of treatment effectiveness in various routine clinical practice settings and in populations not well represented in clinical trials, such as older patients, who may also be more likely to present with comorbidities and not qualify for RCTs. A recently published study using the SEER database demonstrated an improvement in BC‐specific survival after the introduction of CDK4/6is in 2015 in the total SEER population [19]. However, a limitation of the SEER database when not combined with Medicare Claims is the inability to attribute the use of specific treatments to outcomes. By turning to the SEER‐Medicare dataset, which provides access to prescription claims, as is done in the present study, we addressed the question of the association of treatment assignment and OS. The present study complements RWE findings from Goyal et al., who found that CDK4/6is plus ET versus ET alone was associated with a 41% lower risk of death after adjusting for baseline demographic and clinical characteristics (adjusted HR = 0.59 [95% CI, 0.42–0.82]) [23]. Similarly, our multivariable CPH analysis showed a 39% lower risk of death with palbociclib plus an AI versus an AI alone. This similarity was expected as 90% of the patients in Goyal et al. received palbociclib, even though there were differences in study design [23, 53]. Our study focused on patients treated solely with 1L palbociclib and not the CDK4/6i class, included AI as the sole endocrine partner, and was conducted in a more recent version of the SEER‐Medicare database. The new SEER‐Medicare dataset, with two additional years of patient inclusion, allowed for a more recent experience with palbociclib treatment in the clinical setting. sIPTW was used as the primary method to balance patient characteristics to control for confounders, which affect both the outcome and the exposure. This approach approximates randomization in an observational setting and enables us to obtain an mOS.

Comparative OS benefit associated with palbociclib plus an AI in older adults in the United States has been studied using large databases other than SEER‐Medicare (Table S2 contains study details, mOS, and HR values). In the studies conducted by Rugo et al. and Brufsky et al. focusing on older patients aged ≥ 65 and ≥ 75 years, respectively, within the Flatiron Health Analytic Database, palbociclib with an AI was associated with significantly longer OS versus an AI alone, with HRs from sIPTW and PSM analyses ranging from 0.55 (95% CI, 0.42–0.72) to 0.66 (95% CI, 0.51–0.84), respectively [3, 20]. These results are similar to our results despite the SEER‐Medicare population being limited to the de novo population. However, in the DeMichele study of the Flatiron database, the OS HR for patients with de novo mBC was 0.56 (95% CI, 0.40–0.78) using sIPTW [54], while in P Reality X, OS HRs for patients with de novo mBC were 0.68 (95% CI, 0.55–0.84) and 0.77 (95% CI, 0.59–1.00) using sIPTW and PSM analysis, respectively [4]. Each of these HRs is similar to the OS HRs found in this study. Along with OS, these RWE studies also showed prolonged PFS for patients treated with palbociclib plus an AI versus an AI alone [3, 20, 55]. Additionally, a systematic literature review assessing palbociclib treatment outcomes in older patients found that palbociclib combination therapy was effective [56]. Taken together, the evidence from large, multicenter real‐world studies supports the use of palbociclib with an AI for the treatment of HR+/HER2– mBC versus an AI alone in older adults.

Although comparative effectiveness (HRs) was within the range of other studies, mOS was somewhat lower in our study compared with previous RWE studies in older patients (Table S1). sIPTW‐adjusted mOS in patients treated with an AI alone was 25.5 months in our study, compared with 32.4 and 43.4 months in the two Flatiron database analyses, and 34.8 months in the SEER‐Medicare database. Similarly, mOS values in the palbociclib + AI arm in our study were lower than those in Brufsky et al. (mOS was not reached in Rugo et al. and Goyal et al.) [3, 20, 23]. Differences in data source and study design (e.g., variable inclusion and study time frame), and patient baseline and clinical characteristics (e.g., health plan coverage and patient age) could explain these variations in estimates. For instance, when comparing to Goyal et al., who used an earlier iteration of the SEER‐Medicare database, we found more patients with a higher comorbidity burden (NCI comorbidity index score > 0 for 55.0% [AI alone] to 54.4% [palbociclib + AI] after sIPTW versus 32.7% [ET alone] to 30.2% [CDK4/6i + ET]) [23]. The proportion of patients aged ≥ 80 years treated with palbociclib + AI in the sIPTW‐adjusted population of our study was also higher than in Goyal et al. (33.7% vs. 26.6%), which also may have contributed to the differences in mOS estimates between the two studies. Furthermore, our study included data for the year 2020, during which the COVID‐19 pandemic resulted in interruptions in BC screening and start of care, which could negatively affect patient outcomes if delays in screening caused patients to be diagnosed at more advanced stages [57, 58, 59]. Lastly, with over one‐third of the patients being aged ≥ 80 years, it is likely that we are seeing an increase in death due to competing risks. Notwithstanding differences in mOS and patient populations compared with other RWE studies, this study showed that palbociclib + AI was associated with a significant OS benefit compared with AI alone, overall and across subgroups, including age, comorbidity burden, and patients living with liver or lung metastases.

This study has several strengths. Establishing internal validity in RWE studies is important; this study employed statistical methods to balance differences in baseline patient factors and reduce confounding, which could impact survival outcomes; primary (sIPTW) and multiple sensitivity analyses to address baseline differences in covariates as well as additional sensitivity analyses assessing the impact of the start of combination treatment demonstrated consistent OS findings. Our study also fills a demographic gap commonly seen in clinical trials by focusing on older cancer patients (e.g., the median age of patients in the PALOMA‐2 trial was 62 years); cancer incidence is highest in older adults, and approximately half of BC deaths occur in women aged > 70 years [15, 60]. Our results, therefore, add to the effectiveness evidence of palbociclib treatment in an underrepresented population to inform clinical decision‐making [53, 61]. Another strength is our use of the SEER‐Medicare database. The 2023 release of the SEER‐Medicare database has been shown to be generally representative of the majority of elderly patients living in the United States, capturing 35% of the US population [22, 27, 62]. Linkage of Medicare claims with the SEER registry provides confirmatory data on cancer diagnoses along with precise dates, which allows attribution of treatments as specific lines of therapy and substantially minimizes the risk of misclassification. The availability of clinical variables such as tumor grade, metastatic sites at diagnosis, and comorbidities enhanced the set of baseline characteristics on which treatment groups were balanced. Furthermore, almost all (99%) Medicare deaths in the dataset are validated (95% through Social Security Administration data); the vital status information available makes SEER‐Medicare a robust source to examine survival outcomes in older patients with cancer, especially those represented in the US Medicare population [22, 27, 32].

This study also has several limitations, some of which are inherent in observational studies and should be considered when interpreting the findings of this study. This was a retrospective study of a claims database where patients were not randomized to treatments and the rationale for treatment selection was not provided. Although statistical techniques and supplemental sensitivity analyses (sIPTW, PSM, multivariable Cox regression) were implemented to manage selection bias, unobserved confounders may still exist as certain clinical data like Eastern Cooperative Oncology Group performance status, number of metastases, and other social determinants of health relevant to patient access to healthcare (e.g., food security) were not available from SEER‐Medicare. The proportions of patients who were still on 1L treatment or who did not receive 2L treatment indicated longer PFS with palbociclib + AI, in line with PALOMA‐1 and PALOMA‐2; however, PFS is not measured in the SEER‐Medicare. Additionally, inaccurate or missing data were also possible, despite the level of data scrutiny that SEER‐Medicare employs to confirm cancer diagnoses and treatment lines. Furthermore, treatment regimens were defined with a claims‐based algorithm with some inherent risk of misclassification. Although the SEER database has been shown to be generally representative of elderly patients living in the US, capturing 35% of the US population, factors such as geographic area may not be fully captured [22, 25, 63, 64, 65]. While palbociclib is also indicated for patients with BC diagnosed at earlier stages of disease who subsequently become metastatic, this study population was restricted to patients with de novo mBC as SEER does not capture patient progression or metastatic recurrence data; capture of these data in SEER would allow future investigations with more inclusive patient populations [66, 67]. Due to the inherent nature of claims data, where prescribing intent is not verifiable, RWE studies often allow for a time window to define a combination arm where the date of first received treatment constitutes the treatment start (index date). Lastly, in the present study, patients were allowed to receive palbociclib up to 60 days after AI initiation given potential delays in the real‐world setting to receiving treatments, suggesting the possibility that a patient could have died before receiving palbociclib, consequently being assigned to the AI‐alone arm inappropriately. We assessed the potential impact by conducting a sensitivity analysis that explores an extreme scenario where all deaths within 60 days were excluded. Results of this sensitivity analysis were consistent with the main analysis. Also, the likelihood of impact was minimal given the median time from AI to palbociclib start in the combination arms was short at 8 days [64, 65, 68, 69, 70, 71].

## 5 Conclusions

This comparative effectiveness study using the SEER‐Medicare database showed that 1L palbociclib with an AI was associated with an OS benefit versus an AI alone among patients aged ≥ 65 years with de novo HR+/HER2– mBC. Our results add to the growing body of RWE supporting the effectiveness of palbociclib in clinical practice and in an older population historically underrepresented in clinical trials.

## Author Contributions

**Adam M. Brufsky:** conceptualization (equal), investigation (equal), writing – review and editing (equal). **Rickard Sandin:** conceptualization (equal), formal analysis (lead), investigation (equal), methodology (lead), supervision (lead), validation (lead), writing – original draft (lead), writing – review and editing (lead). **Stella Stergiopoulos:** conceptualization (equal), formal analysis (equal), investigation (equal), methodology (lead), supervision (lead), writing – original draft (equal), writing – review and editing (lead). **Connie Chen:** conceptualization (equal), formal analysis (equal), investigation (equal), methodology (equal), writing – original draft (equal), writing – review and editing (equal). **Siddharth Karanth:** formal analysis (equal), investigation (equal), methodology (equal), project administration (lead), writing – original draft (equal), writing – review and editing (equal). **Benjamin Li:** conceptualization (equal), formal analysis (equal), methodology (lead). **Elizabeth Esterberg:** formal analysis (equal), investigation (equal), methodology (equal), writing – original draft (equal), writing – review and editing (equal). **Doris Makari:** conceptualization (equal), methodology (equal), writing – original draft (equal), writing – review and editing (equal). **Sean D. Candrilli:** investigation (equal), methodology (equal), supervision (equal), writing – original draft (equal), writing – review and editing (equal). **Ravi K. Goyal:** conceptualization (equal), formal analysis (equal), investigation (equal), methodology (equal), project administration (equal), supervision (equal), writing – original draft (lead), writing – review and editing (lead). **Hope S. Rugo:** conceptualization (equal), investigation (equal), writing – review and editing (equal).

## Ethics Statement

Upon review of the study material, the RTI International Institutional Review Board deemed this study “not human research” and did not require full review.

## Consent

Patient consent was not required due to use of secondary data from the SEER‐Medicare database and the IRB's determination of this study as “not human research.”

## Conflicts of Interest

S.S., R.S., C.C., B.L., and D.M. are all employees of Pfizer and hold shares and/or stock options. S.K., E.E., S.D.C., and R.K.G. are full‐time employees of RTI Health Solutions, an independent nonprofit research organization, which was a paid consultant to Pfizer in connection with the development of this manuscript. Their compensation is unconnected to the studies on which they work. A.M.B. serves as a consultant for AstraZeneca, Pfizer, Novartis, Lilly, Genentech/Roche, SeaGen, Daiichi Sankyo, Merck, Agendia, Sanofi, Puma, Myriad, Gilead, Epic Biosciences, Blueprint, Caris, and Tempus and provides research support for Agendia and AstraZeneca. H.S.R. serves as a consultant/adviser for Daiichi Sankyo, Mylan/Viartis, NAPO, and Eisai, and reports institutional research support from AstraZeneca, Daiichi Sankyo, F. Hoffmann‐La Roche AG/Genentech, Gilead Sciences, Lilly, Merck & Co., Novartis Pharmaceuticals Corporation, Pfizer, Stemline Therapeutics, OBI Pharma, and Ambryx.

## Supporting information


Data S1.

## Data Availability

The data that support the findings of this study are available from the NCI. Restrictions apply to the accessibility of these data, which were purchased and used under a license/data use agreement for this study.
