# Supplementary material for: Overall Survival With Palbociclib and Aromatase Inhibitor Versus Aromatase Inhibitor Alone in Older Patients With HR+/HER2− Metastatic Breast Cancer
Source: Cancer Med. 2025 Mar 27;14(7):e70719. doi: 10.1002/cam4.70719 (PMC11947744; doi:10.1002/cam4.70719)
Supplement: Supplementary file 1 — Data S1. [file CAM4-14-e70719-s001.docx]

**Overall Survival With Palbociclib and Aromatase Inhibitor Versus Aromatase Inhibitor Alone in Older Patients With HR+/HER2− Metastatic Breast Cancer**

**Supplemental Material**

**Supplemental Table S1. Additional Patient Clinical Characteristics**

|  | **Unweighted** | | | | **Weighted** | | | |
| --- | --- | --- | --- | --- | --- | --- | --- | --- |
|  | **1L AI Monotherapy** | | **1L Palbociclib + AI** | | **1L AI Monotherapy** | | **1L Palbociclib + AI** | |
| **All patients (N, row %)** | **483** | **100.0%** | **296** | **100.0%** | **482** | **100.0%** | **296** | **100.0%** |
| **Surgical and Radiological Intervention^a^** |  |  |  |  |  |  |  |  |
| **SEER-Documented Surgery as Part of Initial Treatment** |  |  |  |  |  |  |  |  |
| Patients with known status | 476 | 98.6% | 289 | 97.6% | 475 | 98.6% | 290 | 97.9% |
| **Surgery performed** | **65** | **13.5%** | **38** | **12.8%** | **66** | **13.8%** | **36** | **12.1%** |
| **SEER-Documented Radiation as Part of Initial Treatment^a^** |  |  |  |  |  |  |  |  |
| Patients with known status | 347 | 71.8% | 207 | 69.9% | 342 | 70.9% | 212 | 71.8% |
| **Radiation administered** | **97** | **20.1%** | **48** | **16.2%** | **99** | **20.6%** | **47** | **16.0%** |
| **Baseline Comorbidities** |  |  |  |  |  |  |  |  |
| **Select Individual components of the NCI index^b^** |  |  |  |  |  |  |  |  |
| Congestive heart failure | 78 | 16.1% | 25 | 8.4% | 66 | 13.7% | 38 | 13.0% |
| Peripheral vascular disease | 51 | 10.6% | 21 | 7.1% | 45 | 9.4% | 27 | 9.1% |
| Cerebrovascular disease | 40 | 8.3% | 17 | 5.7% | 36 | 7.5% | 23 | 7.8% |
| Chronic obstructive pulmonary disease | 84 | 17.4% | 39 | 13.2% | 76 | 15.8% | 51 | 17.3% |
| Diabetes | 135 | 28.0% | 67 | 22.6% | 128 | 26.5% | 78 | 26.3% |
| Diabetes with complications | 50 | 10.4% | 25 | 8.4% | 47 | 9.7% | 31 | 10.6% |

AI = aromatase inhibitors; 1L = first line; NCI = National Cancer Institute; SD = standard deviation.

Note: In compliance with the SEER-Medicare Data Use Agreement, groups with frequencies <11 must be suppressed; therefore, data in some patient groups are collapsed for reporting or suppressed by marking as N/A to prevent derivation of the cell size below 11.

^a^Administered in treatment of the primary tumor

^b^Measured within 6 months before 1L therapy initiation.

**Supplemental Table S2. Comparative US Real-World Studies of 1L CDK4/6i+ET treatment in HR+/HER2– mBC Patients**

|  |  |  |  |  | **mOS** (95% CI), months | |  |
| --- | --- | --- | --- | --- | --- | --- | --- |
| **Study** | **Data Source** | **Age Criterion** | **Treatment Arms** | **Analysis Adjustment Method(s)** | **1L CDK4/6i+ET^a^** | **1L ET alone^a^** | **HR for OS** (95% CI)^b^ |
| Present Study | SEER-Medicare database | ≥65 years | Palbociclib + AI,  AI alone | sIPTW | 37.6 (34.8-42.0) | 25.5 (22.0-28.9) | 0.73 (0.59-0.91) |
|  |  |  |  | PSM | 41.1 (36.6-49.4) | 28.9 (22.0-35.9) | 0.67 (0.54-0.85) |
|  |  |  |  | Unadjusted | 44.0 (37.3-54.5) | 24.2 (20.5-26.7) | 0.54 (0.43-0.66) |
| Goyal et al., 2023 | SEER-Medicare database | ≥65 years | CDK4/6i + ET*,  ET alone | Unadjusted  Adjusted | NE (NE-NE)  NR | 34.8 (29.2-NE)  NR | NR  0.59 (0.42-0.82); *P*<0.001 |
| Rugo et al., 2023 | Flatiron Health Analytic database | ≥65 years | Palbociclib + letrozole,  letrozole alone | sIPTW | NE (NE-NE) | 43.4 (30.0-NE) | 0.55 (0.42-0.72); *P*<0.001 |
| Brufsky et al., 2023 | Flatiron Health Analytic database | ≥75 years | Palbociclib + AI,  AI alone | sIPTW | 43.0 (40.1-NE) | 32.4 (28.2-38.2) | 0.66 (0.51-0.84); *P*=0.0007 |
|  |  |  |  | PSM | 49.0 (40.7-NE) | 37.3 (29.4-44.4) | 0.64 (0.49-0.85); *P*=0.0018 |

1L = first line; AI = aromatase inhibitor; CDK4/6i = Cyclin-dependent kinase 4/6 inhibitor, CI = confidence interval; EHR = electronic health record; ET = endocrine therapy; HR = Hazard Ratio; HR+/HER2– = hormone receptor–positive and human epidermal growth factor receptor 2–negative; mBC = metastatic breast cancer; mOS = median overall survival; NE = not estimable; nIPTW = normalized inverse probability weighting; NR = not reported; PSM = propensity score matching; SEER = Surveillance, Epidemiology, and End Results program; sIPTW = stabilized inverse probability weighting. *90% of patients received palbociclib as the CDK4/6i.^48^

^a^Refer to Treatment arms for specific CDK4/6is and ETs used.

^b^ET alone arm was the reference category for HR estimations.

^c^MD Anderson Cancer Center, Houston, Texas.

**Supplemental Figure S1. Box Plot of (A) Days from mBC Diagnosis to Initiation of 1L Systemic Therapy and (B) Days from Initiation of AI to Initiation of Palbociclib**

A.


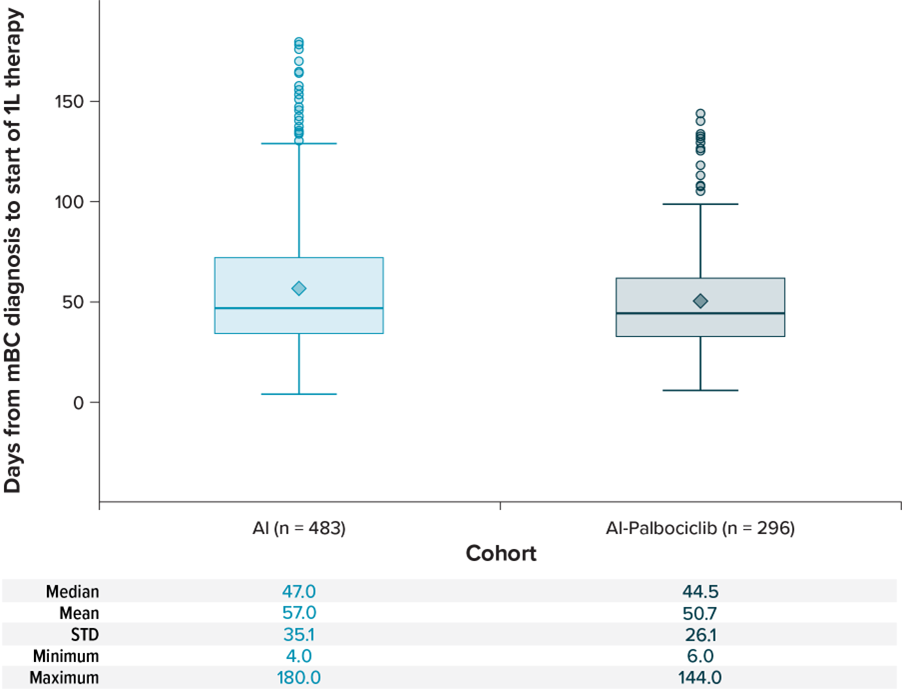


B.


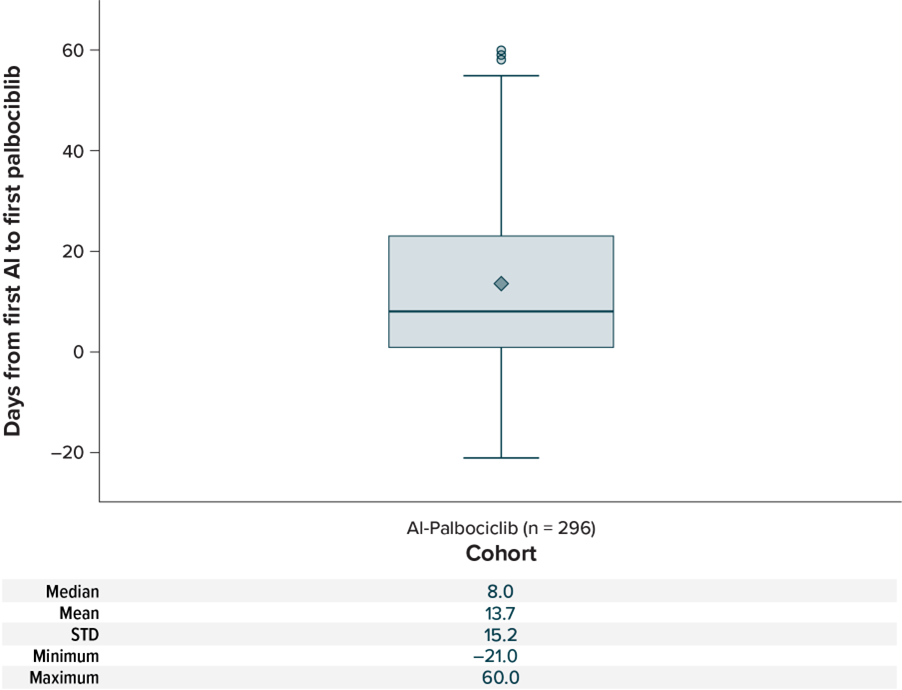


1L = first line; AI = aromatase inhibitor; mBC = metastatic breast cancer; STD = standard deviation.

Note: For panel B, days from first AI to first palbociclib ranged from −21.0 to 60.0. The negative number of days (−21) suggests that palbociclib was initiated before AI.

**Supplemental Figure S2. (A) Unadjusted, (B) sIPTW-Adjusted, and (C) PSM-Adjusted KM Analysis of OS from Start of Palbociclib Treatment**

1. **Unadjusted Analysis**


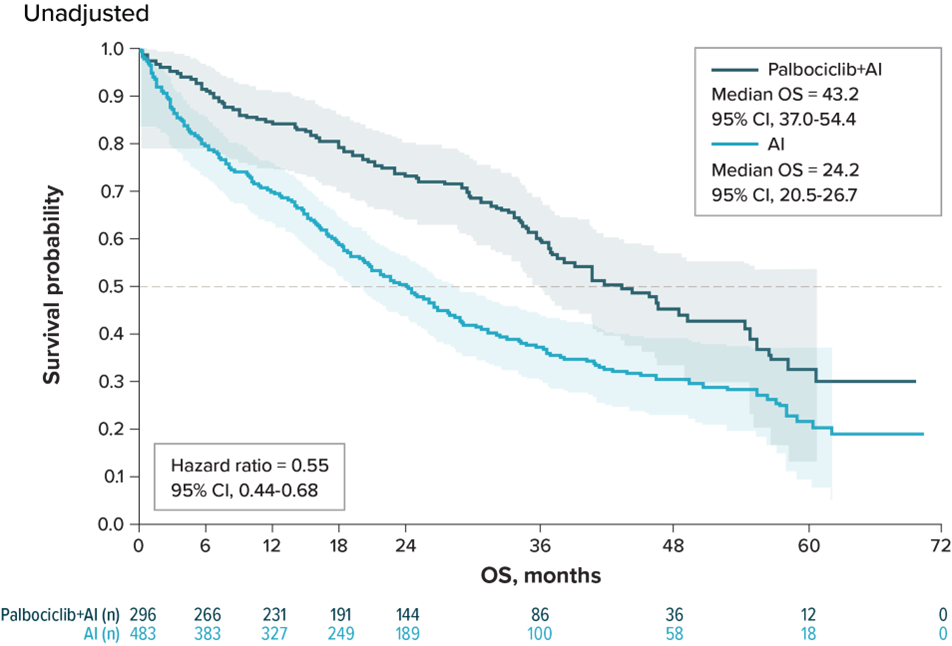


1. **sIPTW Analysis**


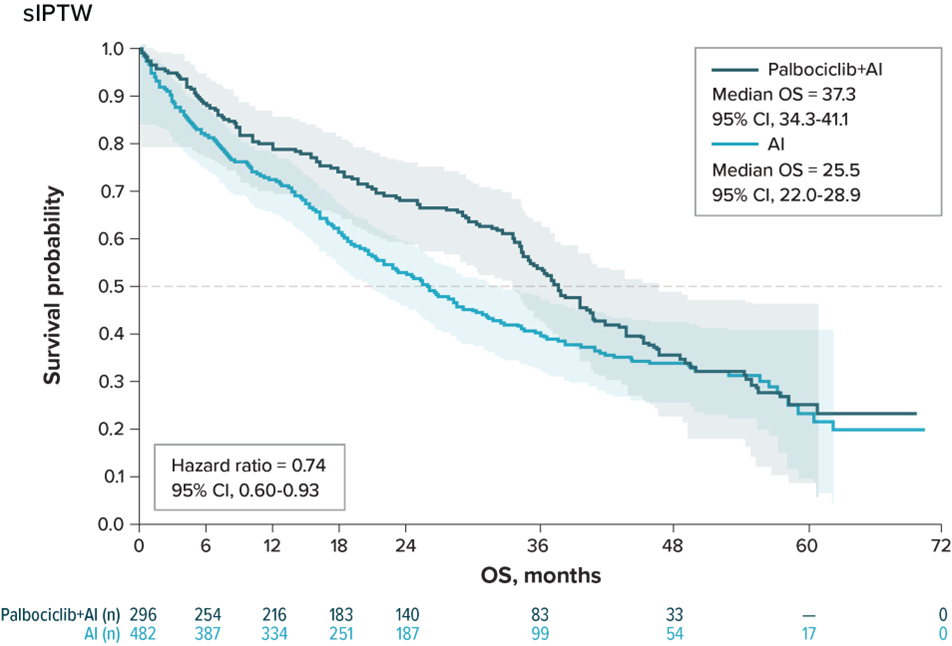


1. **PSM Analysis**


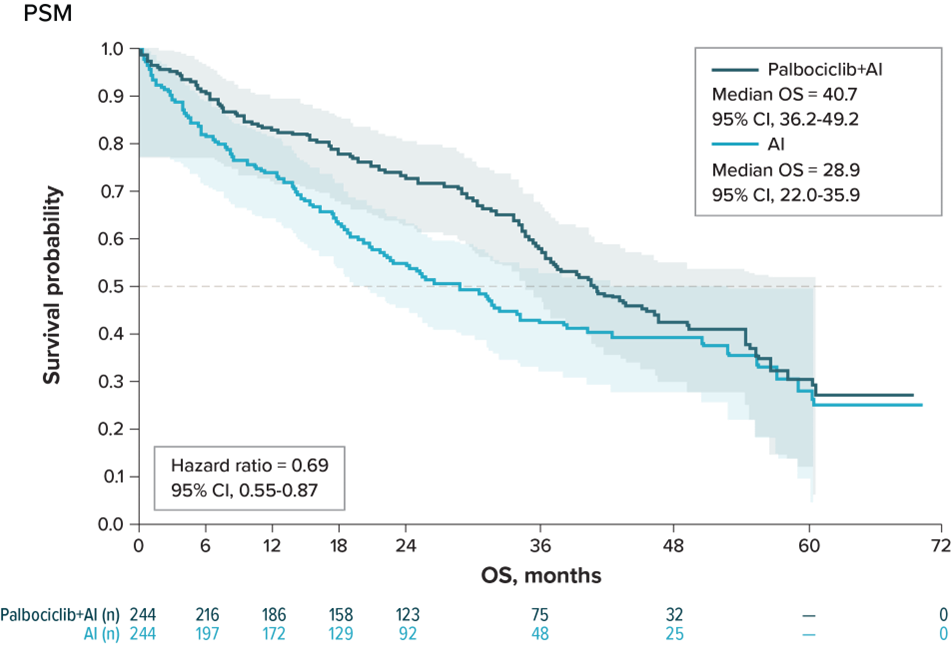


AI = aromatase inhibitor; CI = confidence interval; KM = Kaplan–Meier; sIPTW = stabilized inverse probability of treatment weighting; OS = overall survival.

**Supplemental Figure S3. KM Analysis of OS in Patients who Survived ≥60 Days Following the Index date**

1. **Unadjusted Analysis**


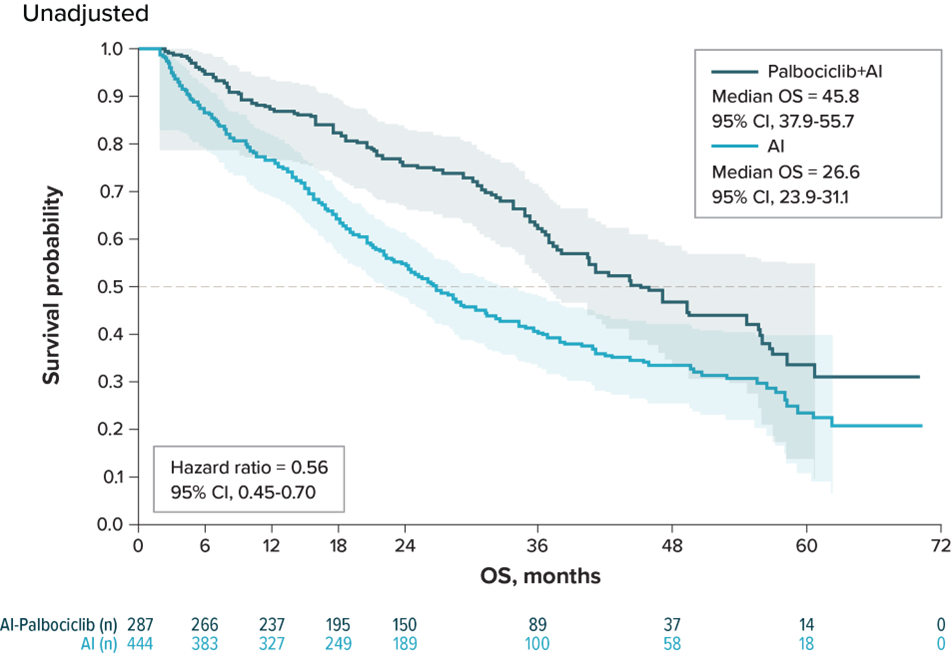


1. **sIPTW Analysis**


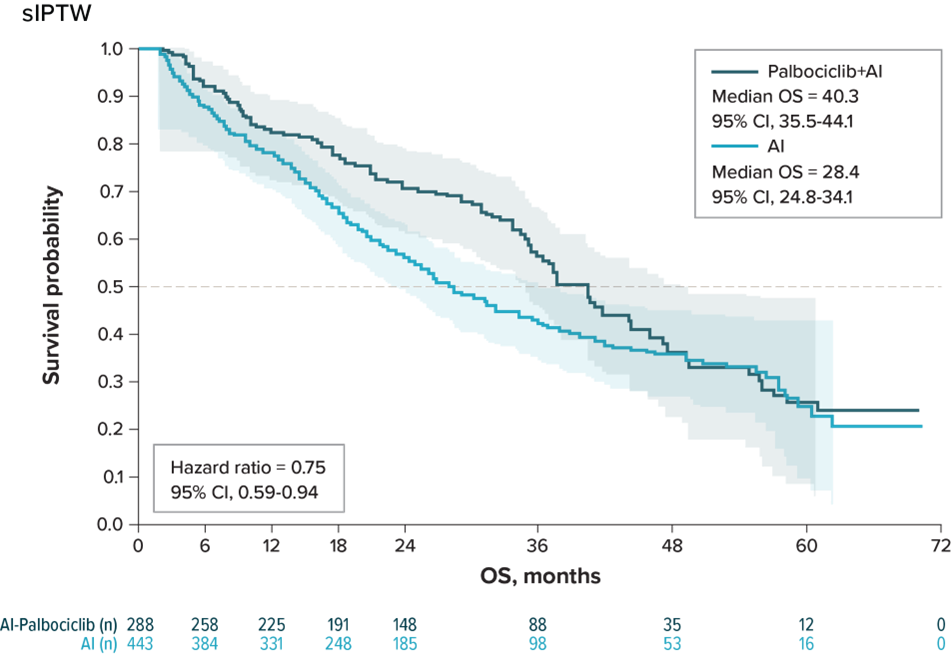


1. **PSM Analysis**


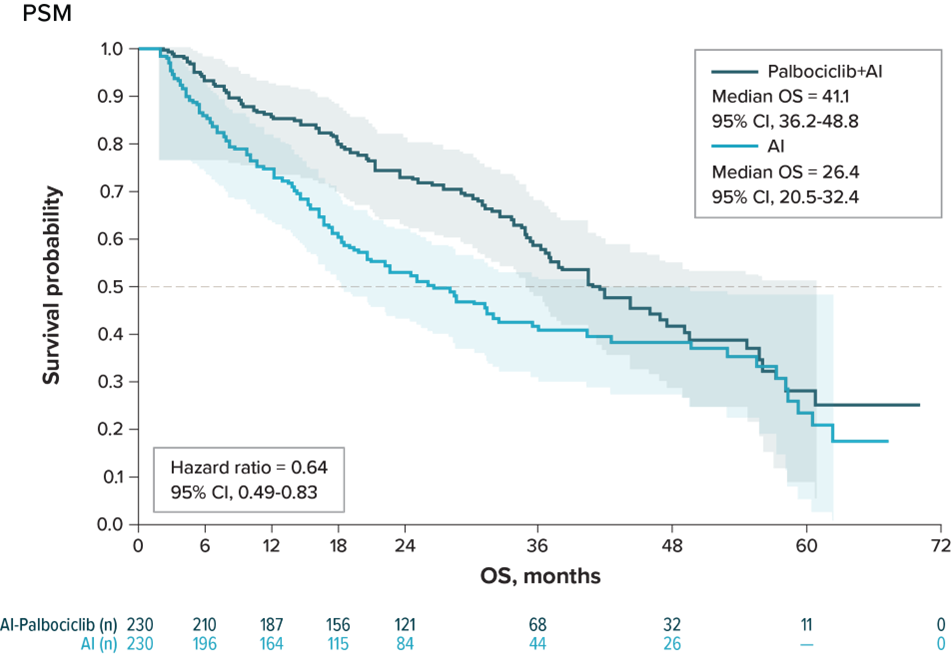


AI = aromatase inhibitor; CI = confidence interval; KM = Kaplan–Meier; sIPTW = stabilized inverse probability of treatment weighting; OS = overall survival; PSM = propensity score matching.
